# Supplementary material for: Persistent red blood cells retain their ability to move in microcapillaries under high levels of oxidative stress
Source: Commun Biol. 2022 Jul 4;5:659. doi: 10.1038/s42003-022-03620-5 (PMC9253111; doi:10.1038/s42003-022-03620-5)
Supplement: Supplementary file 2 — Supplementary Information [file 42003_2022_3620_MOESM2_ESM.pdf]

Supplementary information for

Persistent red blood cells retain their ability to move in microcapillaries under high levels of oxidative stress.

Nadezhda A. Besedina[1*], Elisaveta A. Skverchinskaya[2*], Stanislav V. Shmakov[1], Alexander S. Ivanov[3], Igor V. Mindukshev[2], and Anton S. Bukatin[1,4]

[1]Department of Physics, Alferov University, Saint-Petersburg, Russia; [2]Sechenov Institute of Evolutionary Physiology and Biochemistry, Russian Academy of Sciences, Saint-Petersburg, Russia; [3]Peter the Great St.Petersburg Polytechnic University Saint-Petersburg, Russia; [4]Institute for Analytical Instrumentation of the RAS, Saint-Petersburg, Russia

The Supplementary Information comprises 9 Figures and 2 Movies that can be accessed via the journal webpage.

Supplementary Fig. 1: Images of microchannels on a silicone mold, used for fabrication of microfluidic devices, obtained by scanning electron microscope.

Supplementary Fig. 2: Flow cytometry DCF-DA test for formation of reactive oxygen species.

Supplementary Fig. 3: Proportion of microchannel occlusion cases.

Supplementary Fig. 4: AFM topography of control and treated RBCs.

Supplementary Fig. 5: Flow cytometry EMA test data.

Supplementary Fig. 6: Flow cytometry data in terms of forward and side scattering (FSC/SSC).

Supplementary Fig. 7: Methemoglobin formation under oxidative stress.

Supplementary Fig. 8: AFM maps of control and treated RBCs.

Supplementary Fig. 9: AFM force curves of RBCs, obtained after treatment with different concentration of tBuOOH.

Supplementary Video S1: Representative move of untreated RBCs in a microfluidic channel.

Supplementary Video S2: Representative move of RBCs treated with 1.5 mM *tert*-Butyl hydroperoxide in a microfluidic channel.

Supplementary Data: Source data for graphs

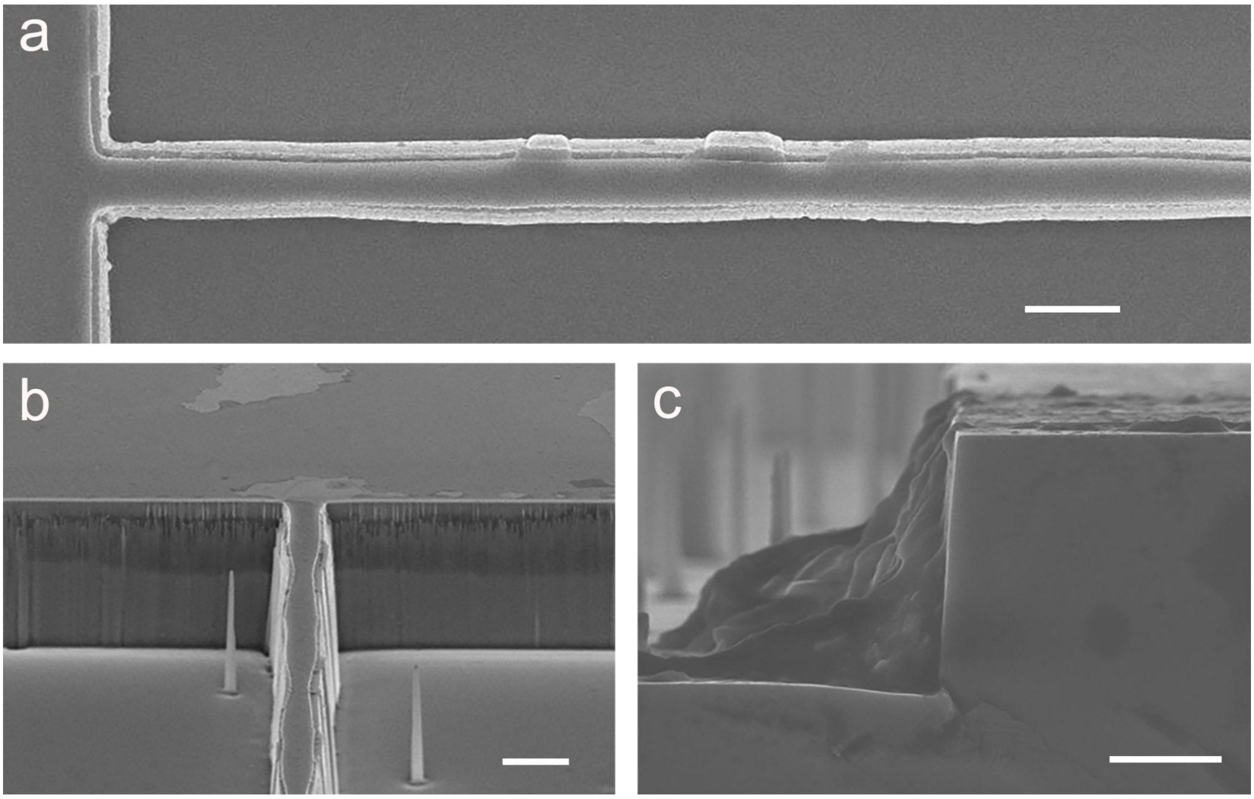

Supplementary Fig. 1 Images of microchannels on a silicone mold, used for fabrication of microfluidic devices, obtained by scanning electron microscope: a) top view, b) projection view, c) side view. The scale bar is 4 μm.

A

DCF-DA	-	-	+	+
tBuOOH	0 mM	0.5 mM	0 mM	0.5 mM

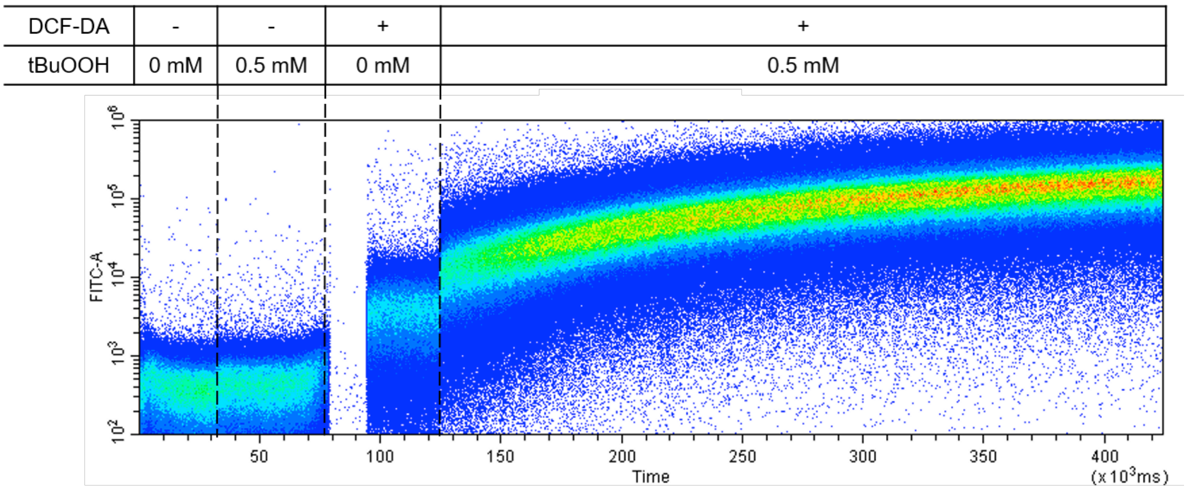

B

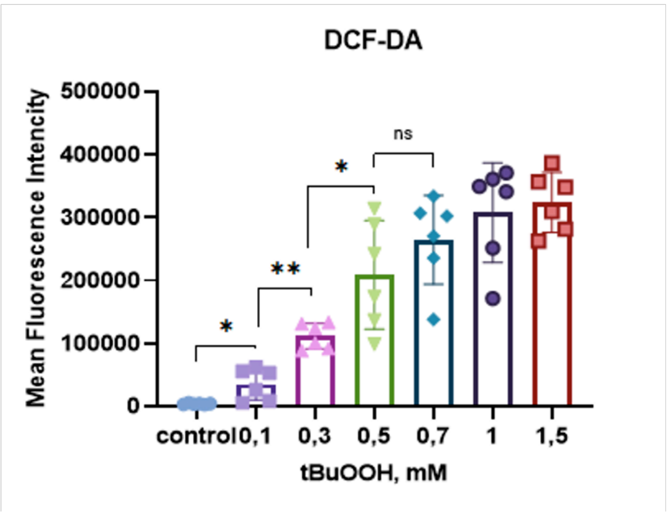

Supplementary Fig. 2 Flow cytometry DCF-DA test for formation of reactive oxygen species. (a) DCF-DA fluorescence intensity over time. The first ~80 seconds show FITC fluorescence intensity of unstained cells, of which the first ~30 seconds - of cells without tBuOOH. Recording of the stained RBCs began at ~95 seconds; at ~123 seconds, 0.5 mM of tBuOOH was added. (b) Mean fluorescence intensity of DCF-DA depending on tBuOOH concentration (n = 5 donors). Data present as mean±SE.

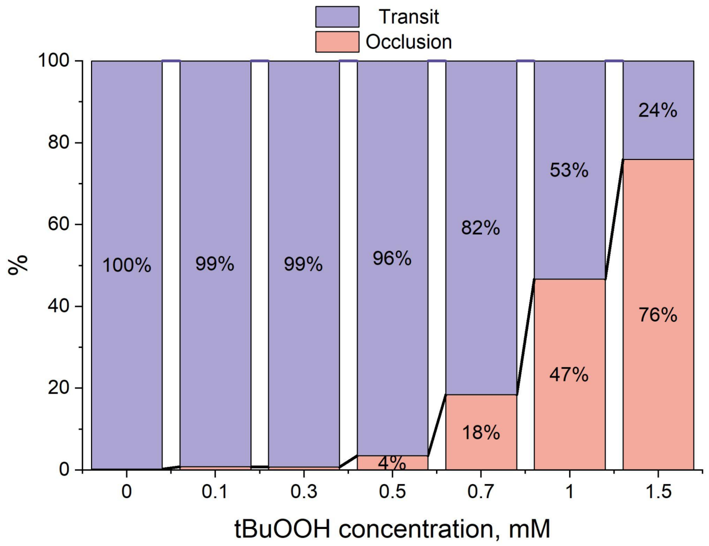

Supplementary Fig. 3 Proportion of microchannel occlusion cases. The higher the concentration of tBuOOH was, the more frequent occlusions appeared. We determined an occlusion as an event in which the RBC stands in front of the channel entrance for ten or more frames (~22 ms). The number of analysed events was n = 11 700 in control, n = 7 000 for tBuOOH 0.1 - 0.7 mM, n = 3 000 for tBuOOH 1 mM and n = 1 500 for tBuOOH 1.5 mM.

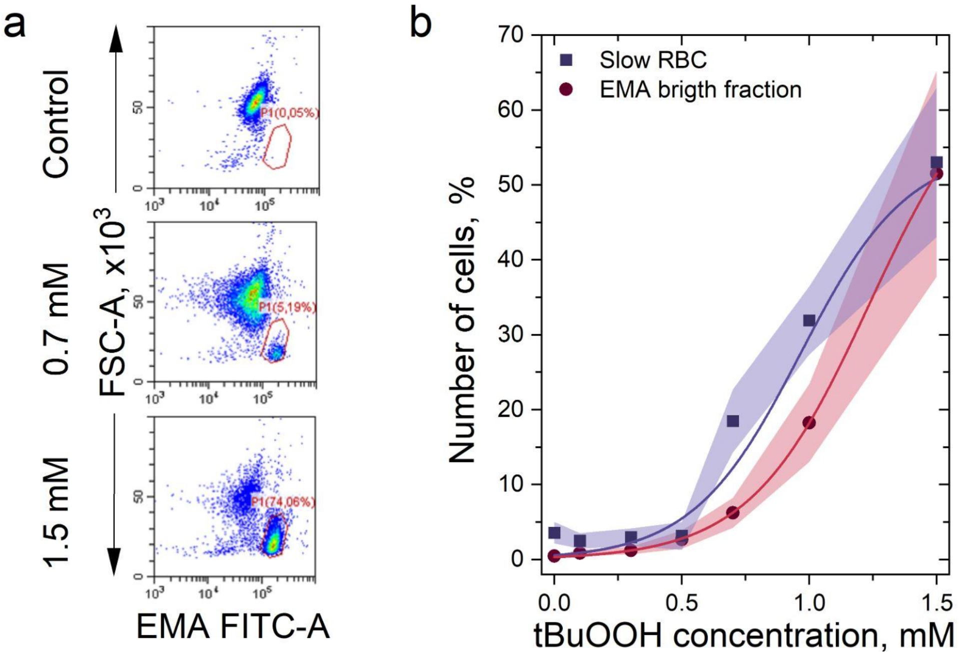

Supplementary Fig. 4 Flow cytometry EMA test data. (a) Dot-plot of EMA-stained RBC in FITC-A/FSC coordinates. One can see much brighter RBCs (red gate). (b) EMA bright RBC fraction depending on concentration of tBuOOH (n = 7 donors) in comparison with slow RBC fraction from microfluidic experiment (n = 5 - 18 donors). Data present as mean±SE, and the error bars are shaded.

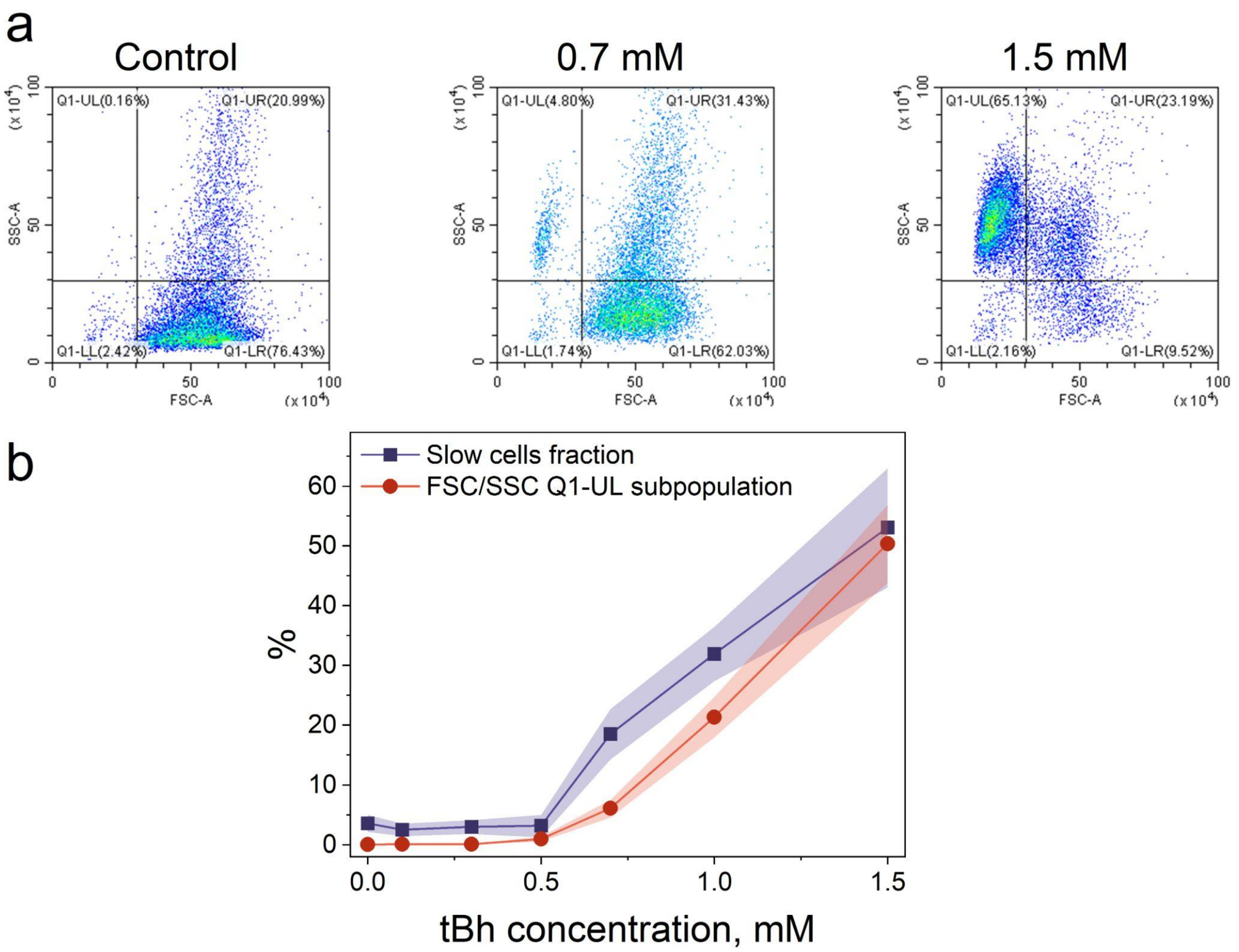

Supplementary Fig 5 Flow cytometry data in terms of forward and side scattering (FSC/SSC). (a) Typical FSC/SSC dot-plot. The Q1-UL quadrant contains damaged RBC subpopulation. Its appearance is recorded at 0.7 mM tBuOOH. This new RBC subpopulation grows with an increase in the tBuOOH concentration. (b) Q1-UL damaged subpopulation proportion in comparison with the slow RBCs. Data present as mean±SE, and the error bars are shaded.

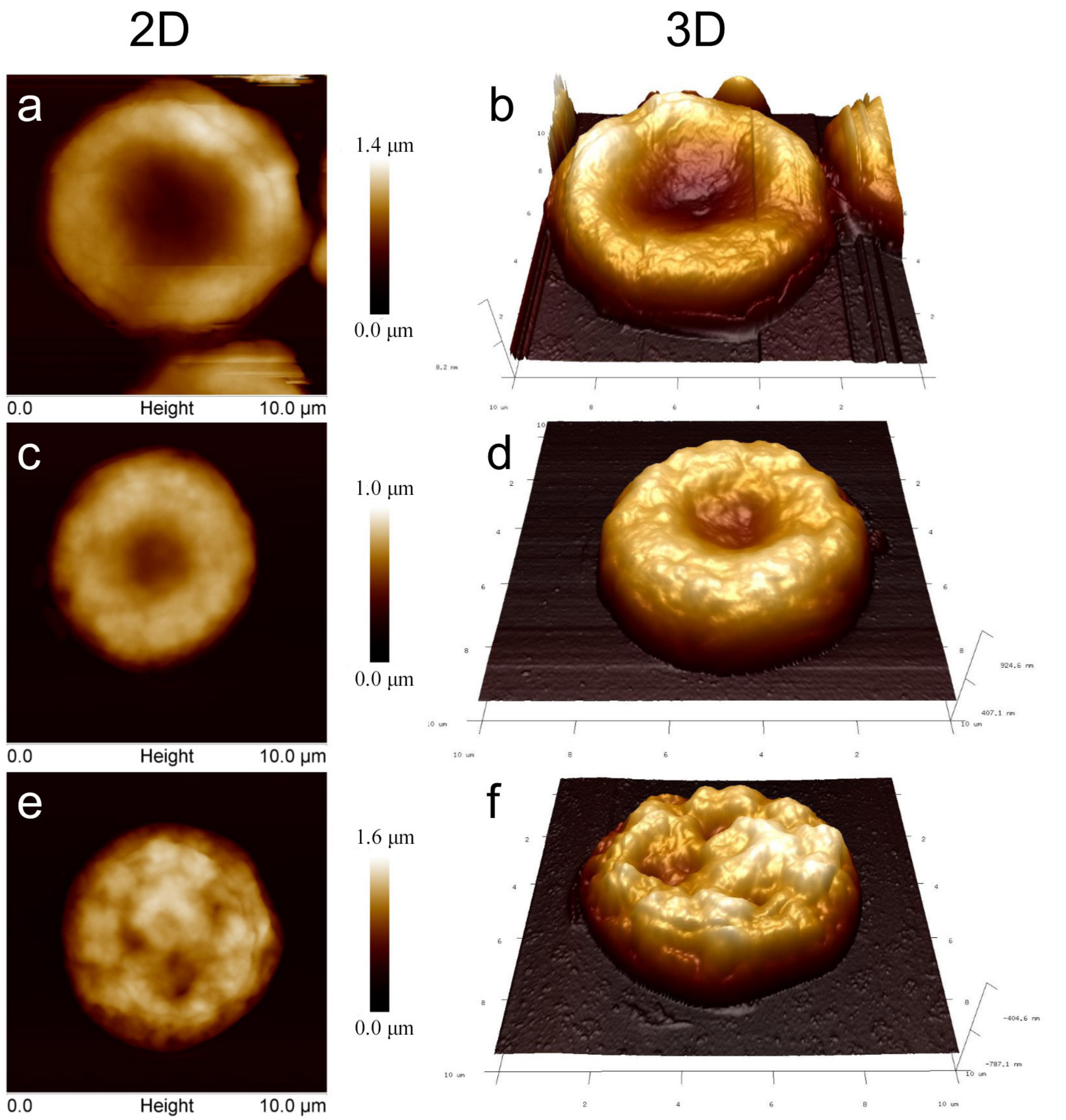

Supplementary Fig. 6 AFM topography showed morphological heterogeneity of treated with tBuOOH RBCs. a, b) 2D and 3D images of control cell topography, respectively; c, d) RBC treated with 1.5 mM of tBuOOH with biconcave disc shape; e, f) RBC treated with 1.5 mM of tBuOOH with spherical shape.

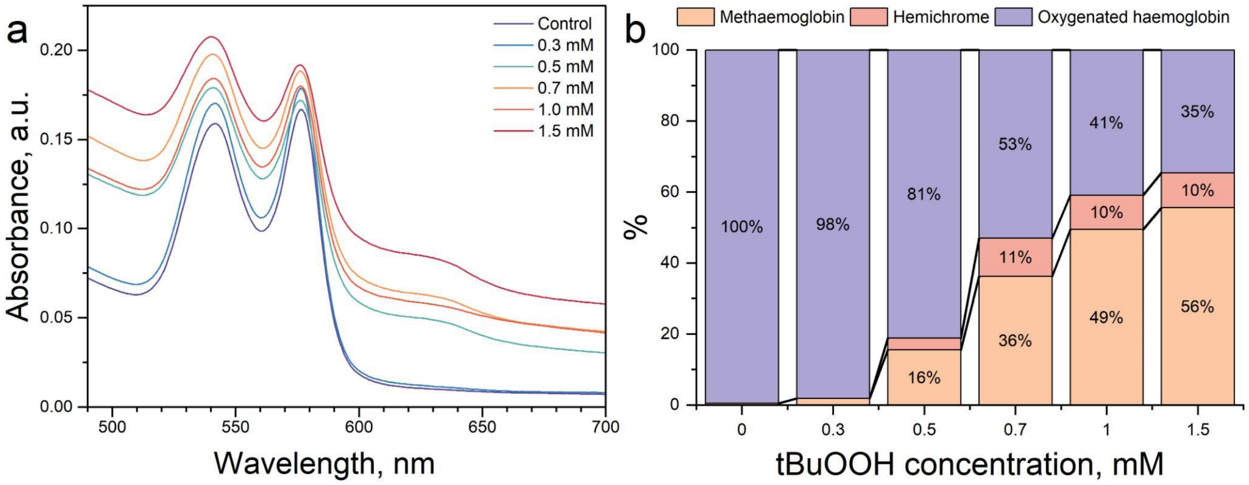

Supplementary Fig. 7 Methemoglobin formation under oxidative stress. a) Representative absorbance spectra. Characteristic peaks in the visible region identify the different oxidation states of hemoglobin. b) Stacked column of hemoglobin species (n = 7 donors).

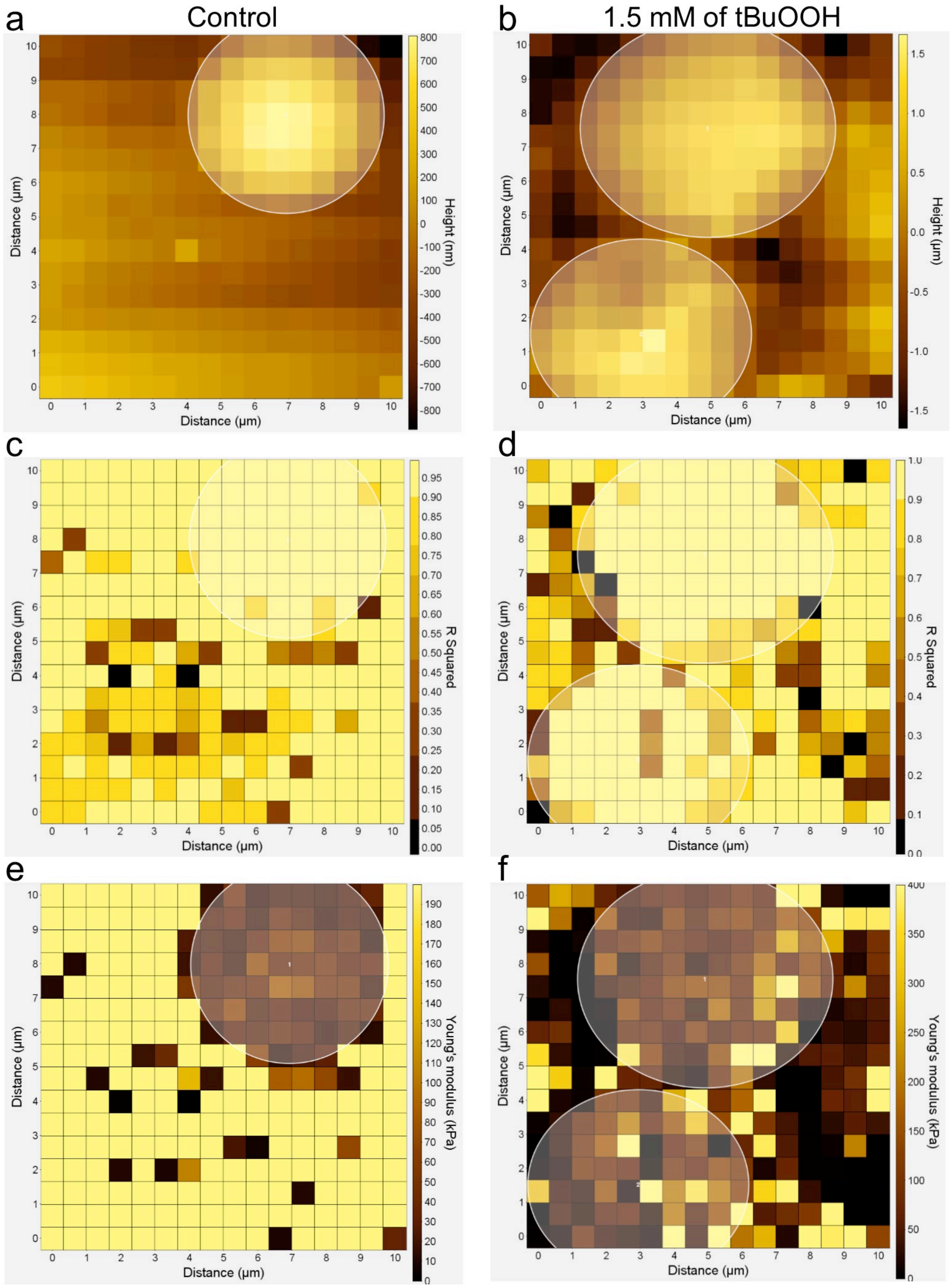

Supplementary Fig. 8 AFM force curve maps. a, b) Maps of samples heights with which the RBCs (white circles) were highlighted; c, d) R^2 maps which show the quality of force curves approximation; e, f) Young's modulus representative maps.

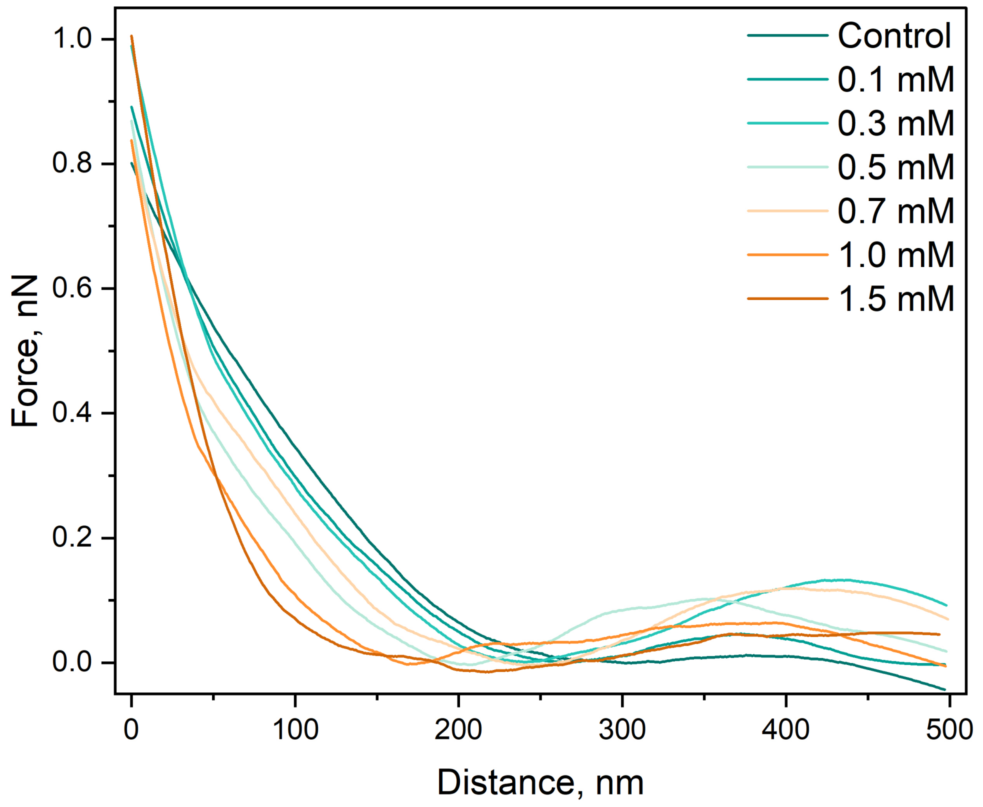

Supplementary Fig. 9 AFM force curves of RBCs, obtained after treatment with different concentration of tBuOOH.
