# Supplementary material for: Persistent red blood cells retain their ability to move in microcapillaries under high levels of oxidative stress
Source: Commun Biol. 2022 Jul 4;5:659. doi: 10.1038/s42003-022-03620-5 (PMC9253111; doi:10.1038/s42003-022-03620-5)
Supplement: Supplementary file 3 — Description of Additional Supplementary Files [file 42003_2022_3620_MOESM3_ESM.pdf]

Description of Additional Supplementary Files

File name: Supplementary Video 1
Description: Representative move of untreated RBCs in a microfluidic channel.

File name: Supplementary Video 2
Description: Representative move of RBCs treated with 1.5 mM tert-Butyl hydroperoxide in a microfluidic channel.

File name: Supplementary Data 1
Description: All the source data for main graphs of the manuscript.
