# Supplementary material for: Persistent red blood cells retain their ability to move in microcapillaries under high levels of oxidative stress
Source: Commun Biol. 2022 Jul 4;5:659. doi: 10.1038/s42003-022-03620-5 (PMC9253111; doi:10.1038/s42003-022-03620-5)
Supplement: Supplementary file 7 — Reporting Summary [file 42003_2022_3620_MOESM7_ESM.pdf]

nature portfolio

Corresponding author(s): Anton S. Bukatin

Last updated by author(s): Nov 2, 2021

Reporting Summary

Nature Portfolio wishes to improve the reproducibility of the work that we publish. This form provides structure for consistency and transparency in reporting. For further information on Nature Portfolio policies, see our Editorial Policies and the Editorial Policy Checklist.

Statistics

For all statistical analyses, confirm that the following items are present in the figure legend, table legend, main text, or Methods section.

n/a	Confirmed	
☐	☒	The exact sample size (*n*) for each experimental group/condition, given as a discrete number and unit of measurement
☐	☒	A statement on whether measurements were taken from distinct samples or whether the same sample was measured repeatedly
☐	☒	The statistical test(s) used AND whether they are one- or two-sided<br>*Only common tests should be described solely by name; describe more complex techniques in the Methods section.*
☒	☐	A description of all covariates tested
☐	☒	A description of any assumptions or corrections, such as tests of normality and adjustment for multiple comparisons
☐	☒	A full description of the statistical parameters including central tendency (e.g. means) or other basic estimates (e.g. regression coefficient) AND variation (e.g. standard deviation) or associated estimates of uncertainty (e.g. confidence intervals)
☐	☒	For null hypothesis testing, the test statistic (e.g. *F*, *t*, *r*) with confidence intervals, effect sizes, degrees of freedom and *P* value noted<br>*Give P values as exact values whenever suitable.*
☒	☐	For Bayesian analysis, information on the choice of priors and Markov chain Monte Carlo settings
☒	☐	For hierarchical and complex designs, identification of the appropriate level for tests and full reporting of outcomes
☒	☐	Estimates of effect sizes (e.g. Cohen's *d*, Pearson's *r*), indicating how they were calculated

Our web collection on statistics for biologists contains articles on many of the points above.

Software and code

Policy information about availability of computer code

Data collection	For microfluidic experiments - XIMEA CamTool<br>For haematology analyzer - no software was used<br>For confocal microscopy - AxioVisionRel. 4.8<br>For AFM - Nanoscope 8.15<br>For flow cytometry experiments - CytExpert 2.4 (CytoFLEX, BC) and BD FACSDiva 9<br>For SEM - SmartSEM<br>For absobance spectroscopy - software of SPECS SSP-715-M (Spectroscopic systems, LTD, Moscow, Russua).
Data analysis	For microfluidic experiments - custom script in MATLAB R2014a, OriginPro 2021b<br>For haematology analyzer - Microsoft Exel 2016, OriginPro 2021b, for digitized histogram - WebPlotDigitizer<br>For confocal microscopy - AxioVisionRel. 4.8, OriginPro 2021b<br>For AFM mechanical properties measurements - AtomicJ 2.2, OriginPro 2021b<br>For AFM imaging - NanoScope Analysis<br>For flow cytometry experiments - CytExpert 2.4, BD FACSDiva 9, Microsoft Exel 2016, OriginPro 2021b<br>For absobance spectroscopy - Microsoft Exel 2016

For manuscripts utilizing custom algorithms or software that are central to the research but not yet described in published literature, software must be made available to editors and reviewers. We strongly encourage code deposition in a community repository (e.g. GitHub). See the Nature Portfolio guidelines for submitting code & software for further information.

Data

Policy information about availability of data

All manuscripts must include a data availability statement. This statement should provide the following information, where applicable:

- Accession codes, unique identifiers, or web links for publicly available datasets
- A description of any restrictions on data availability
- For clinical datasets or third party data, please ensure that the statement adheres to our policy

The datasets generated during and/or analysed during the current study are available from the corresponding author on reasonable request.

Field-specific reporting

Please select the one below that is the best fit for your research. If you are not sure, read the appropriate sections before making your selection.

☒ Life sciences ☐ Behavioural & social sciences ☐ Ecological, evolutionary & environmental sciences

For a reference copy of the document with all sections, see nature.com/documents/nr-reporting-summary-flat.pdf

Life sciences study design

All studies must disclose on these points even when the disclosure is negative.

Sample size	Our research includes data from 18 healthy volunteers. To calculate sample size, we used data on erythrocyte volume change (MCV, n=18), a key indicator for microchannel transit capability; and assessment of erythrocyte viability under oxidative stress (calcein-am test is a rapid and accurate method to measure cell viability and cytotoxicity, n=7); as well as our previous studies. The calculation was performed at the selected power level of analysis 0.8, using a publicly available calculator https://www.stat.ubc.ca/~rollin/stats/ssize/n2.html
Data exclusions	All the obtained data were included in the analysis.
Replication	The reproducibility of the results is confirmed by the normality of the distribution (Gaussian).
Randomization	There was no special selection for volunteers.
Blinding	The study did not require blind assignment to groups since there were no starting groups; all samples received were treated equally.

Reporting for specific materials, systems and methods

We require information from authors about some types of materials, experimental systems and methods used in many studies. Here, indicate whether each material, system or method listed is relevant to your study. If you are not sure if a list item applies to your research, read the appropriate section before selecting a response.

Materials & experimental systems

n/a	Involved in the study	
☒	☐	Antibodies
☒	☐	Eukaryotic cell lines
☒	☐	Palaeontology and archaeology
☒	☐	Animals and other organisms
☐	☒	Human research participants
☒	☐	Clinical data
☒	☐	Dual use research of concern

Methods

n/a	Involved in the study	
☒	☐	ChIP-seq
☐	☒	Flow cytometry
☒	☐	MRI-based neuroimaging

Human research participants

Policy information about studies involving human research participants

Population characteristics	Material for the study was obtained from 18 volunteers of both sexes (11 women and 7 men), ages 22-68.
Recruitment	All volunteers involved had no health problems.
Ethics oversight	The study was conducted according to the guidelines of the Declaration of Helsinki and in accordance Convention on Human Rights and Biomedicine (ETS No 164) and approved by the Institutional Ethics Committee of Sechenov Institute of Evolutionary Physiology and Biochemistry (protocol code N2-02, 2021-02-26). Informed consent was obtained from all subjects involved in the study. Written informed consent was obtained from the volunteers for blood collection. The data

were anonymized, so consent for publication was not required. All samples of blood components after use were destroyed according to the protocol of disposal of biological materials (N2.1.7.2790-10 of December 12, 2010 "Sanitary and epidemiological requirements for the treatment of medical waste", class B (blood and body fluids).

Note that full information on the approval of the study protocol must also be provided in the manuscript.

Flow Cytometry

Plots

Confirm that:

☒ The axis labels state the marker and fluorochrome used (e.g. CD4-FITC).

☒ The axis scales are clearly visible. Include numbers along axes only for bottom left plot of group (a 'group' is an analysis of identical markers).

☒ All plots are contour plots with outliers or pseudocolor plots.

☒ A numerical value for number of cells or percentage (with statistics) is provided.

Methodology

Sample preparation	Blood was collected by venipuncture in S-monovette tubes. After first centrifugation, plasma and buffy-coat layer were removed, and RBCs were washed twice and resuspended in isotonic buffer. Oxidative stress was induced by tBuOOH (0.1, 0.3, 0.5, 0.7, 1, and 1.5 mM), during 4-5 h, 37 °C on Thermoshaker. Our previous study showed that it is crucial to fixate the ratio [tBuOOH]/[RBC] to acquire correct results of oxidative stress effects on RBCs. Therefore, in all experiments, the concentration of RBCs in incubation suspension was kept constant.
Instrument	CytoFLEX B4-R2-V0 and BD FACSCanto
Software	CytExpert 2.4 and BD FACSDiva 9
Cell population abundance	Blood was collected by venipuncture in S-monovette tubes. After first centrifugation, plasma and buffy-coat layer were removed, and RBCs were washed twice and resuspended in isotonic buffer. Leukoreduction was not performed. In all the experiments 20 000 events were collected.
Gating strategy	For determining starting FSC/SSC position we used CytExpert autogating in all experiments. In annexin-V experiments, annexin-V negative populations were gated using unstained controls. In the Annexin-V test, we gated the cells on the histogram plot to the right of the negative (control) population. The EMA-brigth population was gated in FSC-A/FITC-A coordinates.

☒ Tick this box to confirm that a figure exemplifying the gating strategy is provided in the Supplementary Information.
